# Palliative Oncological Patients with Insomnia: Concerns of the Patients and Their Relatives’ Perception

**DOI:** 10.3390/ijerph18168509

**Published:** 2021-08-12

**Authors:** Antoni Font Guiteras, Helena Villar Abelló, José Planas Domingo, Cristina Farriols Danés, Ada Ruiz Ripoll, Rita Berger

**Affiliations:** 1 Department de Psicologia Bàsica, Evolutiva i de la Educació, Universitat Autònoma de Barcelona, 08193 Barcelona, Spain; Antonio.font@uab.cat (A.F.G.); villar.helena@gmail.com (H.V.A.); 2 Palliative Care Unit, Geriatric Department, Centre Fòrum, Hospital del Mar, 08003 Barcelona, Spain; jplanas@parcdesalutmar.cat (J.P.D.); CFarriols@parcdesalutmar.cat (C.F.D.); 94784@parcdesalutmar.cat (A.R.R.); 3 Departamento de Psicología Social y Cuantitativa, Universitat de Barcelona, 08035 Barcelona, Spain

**Keywords:** palliative care, insomnia, concerns, terminal cancer, primary caregiver

## Abstract

Insomnia is one of the most frequent symptoms and usually generates significant stress in 60% of patients with advanced cancer. Worries from the patients’ and relatives’ perspective are crucial to improve the patients’ quality of life but have received limited attention. The aims were to identify the concerns of patients with insomnia in the terminal illness stage in a palliative care unit and the relatives’ perception, and to compare both. Here, 63 patients and 53 relatives answered a questionnaire about worries in the personal, spiritual, family-related and economic area, as well as a quality-of-life uniscale. The results showed that the relatives’ most frequent concern was “Having lived life to the fullest” (100%), and the most intense was “The possible suffering during the process” (9.2/10). The patients’ most expressed concern was: “Having unfinished business” (100%), and the most intense was “Suffering during the process” (9.3/10). Quality of life showed an average value of 6.95 out of 10. Relatives only coincided significantly in: “Not knowing what happens after death” (r = 0.600; *p* = 0.000). These results bring visibility to concerns during the final stage of oncological palliative patients with insomnia from the patients’ and relatives’ perspective. Knowing both is useful for professionals to foster the well-being for a short, yet very important, period for patients, relatives and the caregiving team.

## 1. Introduction

Oncological patients in advanced stages face a progressive incurable disease with limited response to treatments, and often seem to present severe and changing symptoms during the final stage of life. The incidence of various physical symptoms ranges between 8 and 10 per patient [1]. This can generate an emotional impact on the patient, the family, and the caregiving team, causing significant changes at physical, emotional, social, and spiritual levels. In fact, 25–50% of oncological patients in advanced stages present psychiatric morbidity predominantly with depression and anxiety disorders [2], and ruminations and concerns [3,4]. It was observed that insomnia, one of the most frequent symptoms [5], increases before death [6] and affects about at least one of three palliative care patients [7], and it usually generates significant stress in 40–60% of patients with advanced cancer [5,8]. However, it has received a limited amount of attention [7], bearing in mind that it contributes doubly to the patient’s discomfort. On the one hand, it generates concern when it is interpreted as a deterioration or a loss of vital reparative capacity. On the other hand, it allows an increase in time and intensity of the worries that the terminal condition carries, in a frequent situation of loneliness. Thus, the insomnia patient’s distress can increase emotional discomfort, reducing the quality of life and the negative experience of other symptoms [8,9,10,11].

According to the Spanish Society for Palliative Care, the terminally ill patient’s needs must be addressed efficiently, with the objectives of improving comfort and quality of life, while respecting the families’ and patients’ viewpoints, and in accordance with their values, preferences, and beliefs [12]. A recent review about the experiences of patients and caregivers at the end of life showed that evidence for enhancing hospice care experiences is still very limited [13]. Previous studies have indicated that there are many unmet needs in patients with cancer, the most prevalent being psychological support and the need for information [14]. In patients with advanced cancer, research that focuses on their subjective needs (not only physical ones) is still scarce [15,16,17,18,19]. Not providing the necessary attention for the needs of the patients and their families (from their point of view) contributes to an increase in discomfort [20]. At the same time, it supposes a less effective use of the medical care and economic resources, given that the concerns of the sick and their relatives can have an influence on each other, determining, at least in part, the patient’s quality of life during the terminal phase [21,22,23,24]. In the context of chronic disorders, palliative care service units have the need to offer a service centred on the basic physical and psycho-social needs and concerns of the patients to improve the advanced cancer patients’ quality at the short end of life [25]. Communication in palliative care is therefore very important for clinicians, patients, and relatives, but there are many obstacles to be overcome [26,27,28]. At the end-of-life, patients with advanced cancer are often not able to effectively communicate their needs to the palliative care service [25]. Family members here play an important role—relatives who receive a psychoeducational intervention on the patient’s situation improve their capability of being able to take care of the sick, and this also helps to reduce the patient’s levels of depression and discomfort [29,30]. Moreover, the demands of the patients and relatives towards medical personnel are also conditioned by these mutual influences [31,32]. An important, but not yet adequately researched, aspect is the perception of the patient’s needs that the relatives have. This perception has a direct influence on the interaction of relatives with the patient and on the demand for support from the professional. It is therefore important that research examines the relatives’ perception of the needs and concerns of patients and deepens this knowledge. Furthermore, having information on the concerns of the oncological palliative patient with insomnia from the patients’ and relatives’ perspectives is relevant for providing the best possible attention specifically for these patients and situations characterised by short life expectancy and changing needs. Research on the concordance or not of patients’ concerns and its perception by their relatives is scarce [8]. Investigating the concerns that patients have is transcendental for dying in peace, and there is little time to identify and to attend these needs. Thus, the caregivers must know (perceive adequately) these needs to reassure the patient.

Summarising, previous studies have shown several research gaps: evidence for enhancing hospice care experiences is very limited; research about the required information on the subjective needs in patients with advanced cancer is just as rare as studies on family members’ perception of patients’ needs [8] but is needed as patients often cannot communicate their needs efficiently and need their relatives for this communication. The present study therefore aimed to analyse the oncological patients’ needs at the end of their lives and their relatives’ points of view (specifically of the primary caregiver) surrounding these concerns in a particularly stressful situation, those of patients with insomnia.

This research aimed to fill in one of these gaps by contributing to the body of literature in three ways: (a) it describes and therefore increases existing knowledge on the worries of an advanced oncological patient with insomnia, (b) identifies and defines the perception of the primary caregiver about the patient’s concerns, and (c) compares the patient’s concerns with the perception of these concerns by the relative giving new insight into both, which provides valuable information for specific therapeutic interventions that can reduce the worries of an advanced patient with insomnia.

## 2. Materials and Methods

### 2.1. Participants and Procedure

The analysed sample consisted of 63 patients with terminal oncological disease (43 men and 20 women) hospitalised in the Palliative Care Unit of the Esperança Hospital (Public Health Centre of the Mar Hospital in Barcelona), and one family member per patient, comprising 53 relatives (14 men and 39 women). In three of the cases, there was no available relative, and in seven others, the relatives were not present at the time of assessment (*n* = 116). The study was approved by the Ethical Committee of the hospital Mar Health Park. The inclusion criteria were scoring a Karnofsky performance status greater than 30, being of legal age, and having moderate or severe insomnia. To diagnose and select patients with insomnia, the medical team applied the Sleep Disturbance Scale (SDS) [33,34] at the time of the clinical assessment, and recorded a maximum of 4 errors on the Pfeiffer test (having considered an appropriate cognitive level to be able to understand and participate in the survey). The SDS was chosen for several reasons: it is a short measure that identifies insomnia in the previous 24 h. Furthermore, it was successfully applied in similar settings [33,34]. During the data collection period, there were 550 patients in the care unit (335 men and 215 women). Out of these, those diagnosed with insomnia by healthcare professionals during the period and had also met the inclusion data criteria became a part of the study (*n* = 74). We excluded 6 cases due to deteriorating conditions or death before the interviews took place, and 5 others were excluded for rejecting the participation in the survey (6.75%). First, each patient was requested to fill the informed consent form, after having read the information sheet of the study. A psychologist asked the patients individually in their room of the Palliative Care Unit, using a questionnaire with nine items on the needs and worries and one item on the subjective quality of life. Additionally, each patient explained in detail their concerns during an open interview. Furthermore, each relative was asked to fill in the corresponding questionnaires regarding the needs and worries of the patient. Finally, the relatives were interviewed without the presence of the patient about detailed patients’ concerns using the same procedure. The interviews with patients and relatives lasted approximately 20 min in each case.

### 2.2. Instruments

#### 2.2.1. Medical and Sociodemographic Data Sheet

The following data of the patient were collected through their medical record: gender, age, origin, cancer location, tumour extension (according to ICD-9-CM), the effect on the CNS and the beginning of the insomnia disorder.

#### 2.2.2. Patient Concerns

Patients answered to a reduced version of the 2010 Buscemi et al. [21] questionnaire “Needs and concerns of the patient in cancer palliative care”. We chose this questionnaire of concerns because we have previously used it successfully in the context of terminal patients to identify undetected needs and concerns. They answered the following nine items of concern from the questionnaire, rating each item on a scale from 0 to 10: “Not knowing what happens after death”, “What will happen to my family afterwards”, “Fear of being forgotten”, “Having unfinished business”, “The possible suffering during the process”, “The possibility of dying alone”, “Being able to say goodbye to loved ones”, “Having had a positive effect on others”, and “Having lived life to the fullest”. The questionnaire was applied individually and without the presence of the relative.

#### 2.2.3. Subjective Quality of Life

A visual analogue uniscale (VAS), ranging from 0 to 10, in which 0 represents “My life this past week has been normal” and 10 “My life this past week has been extremely unsatisfying due to my health” was completed by the patient [35], similarly to other studies with terminally ill patients [36,37].

#### 2.2.4. Patient’s Concerns Perceived by the Relative

Relatives answered the same questionnaire as the patients did (“patient’s concerns”), but in this case, the questions were about the degree of worry they believed their patient would have about each proposed aspect, ranging from 0 (not at all worried) to 10 (extremely worried). For example: “How much do you believe that the patient is worried about the possible suffering during the process”. The questionnaire was applied individually and without the presence of the patient.

#### 2.2.5. Open Interviews with the Patients and with the Relatives

Additionally, to complete the previous information, patients and relatives participated in an open interview about the patients’ concerns and needs performed by a psychologist to obtain a detailed and deeper insight. The open interview took about 20 min per patient. All the patients gave open answers about worries related to their situation. Subsequently, this qualitative information was transcribed literally. Two other researchers placed each expression of concern in one of the following categories: family concerns, financial concerns, and spiritual concerns. In the event of a mismatch, two more researchers were asked, until a consensus of at least three researchers was reached. Thus, each worry could be placed in one of the mentioned categories. Collection and analyses of these qualitative data based on the views of the participants were guided by the grounded theory method [38].

### 2.3. Statistical Analysis

Statistical analysis was based on quantitative and qualitative data. Quantitative data analysis was carried out with Windows IBM SPSS version 22.0 Armonk, NY, USA, IBM Corp. Descriptive statistical methods were used for the survey data related to the needs and concerns of patients and relatives, the patients’ quality of life, and sociodemographic data. For the quantitative patient–relative analysis, due to the nonnormality of the variables according to the applicability of the Shapiro–Wilk test, Spearman correlations were used. For the qualitative study (interviews) of the variables of the types of concerns and needs expressed by the patient, a content analysis was carried out by two researchers independently to identify the elements of the studied reality and synthesise the knowledge into categories from the collected qualitative information.

## 3. Results

### 3.1. Sociodemographic and Clinical Data of the Participants

The age range of the patients in this sample study ranged between 52 and 91 years of age (average = 74.4; SD = 11.7); 68.3% were men and 31.7% were women. The majority came from home (79.4%) and the most prevalent location of tumours were the lungs (22.2%), urinary bladder (15.9%), and colon (14.3%). In addition, 81% had a dissemination of the illness and, in 90.5% of the cases, it had not extended to the brain. Furthermore, 39.7% suffered insomnia since before the diagnosis of the oncological illness, another 39.7% since the diagnosis, and the remaining 20.6% since going into hospital. The patient’s medical and sociodemographic data are shown in Table 1.

### 3.2. Insomnia and Patient Concerns

We found that 87.5% of the patients stated having nocturnal concerns which generated difficulties in falling or staying asleep. Personal (present in 95.2% of cases) and family (66.6%) worries predominated, followed by spiritual (30.2%) and economic ones (25.4%). The most expressed concerns by the patients were: “Having unfinished business” (*n* = 63, 100%), “The possible suffering during the process” (*n* = 59, 93.6%), “What will happen to my family afterwards” (*n* = 58, 92%) and “The possibility of dying alone” (*n* = 57, 90.5%). Additionally, the most intense ones were “The possible suffering during the process” with an average of 9.3 points out of 10. “What will happen to my family afterwards” had 8.4 points and “Fear of being forgotten” had 8.2 (see Table 2).

The various concerns gathered from conversations with the patient were grouped into personal, family, spiritual, and economic concerns (see Table 3). The most frequent personal-related concerns were “The loss of autonomy” (*n* = 59, 93.7%). Regarding family-related concerns, it was “How will my family cope after the loss” (*n* = 26, 41.3%). Regarding spiritual concerns, it was “The need to love and be loved” (*n* = 12, 19%), and regarding economic concerns, it was “The current financial situation” (*n* = 12, 19%).

### 3.3. Patient Concerns according to the Relative

According to the relatives, the patient’s most frequent concerns were: “Having lived life to the fullest” (*n* = 53, 100%), “What will happen to my family afterwards” (*n* = 50, 94.3%) and “Having had a positive effect on others” (*n* = 43, 81.1%). In addition, in their view, the patient’s most intense concerns were “The possible suffering during the process” with 9.2 points, “What will happen to my family afterwards” averaging 8.4, “Being able to say goodbye to loved ones” with an average of 7.5, followed by “Not knowing what happens after death” and “Having unfinished business”, both with a 7.3 score, always on a 10-point scale (see Table 2).

To analyse the possible relationship between the concerns of each specific patient related to their main carer’s perception of these, Spearman correlations were carried out. Significant and positive correlations were observed in the following concerns: “Not knowing what happens after death” r = 0.54 (*p* = 0.002), “What will happen to my family afterwards” r = 0.34 (*p* = 0.012), “Having unfinished business” r = 0.42 (*p* = 0.007), “The possible suffering during the process” r = 0.57 (*p* = 0.001), “Having lived life to the fullest” r = 0.43 (*p* = 0.003) and the concern “Fear of being forgotten” r = 0.45 (*p* = 0.03). No other significant correlations were observed regarding the rest of the items: “The possibility of dying alone”, “Being able to say goodbye to loved ones” and “Having had a positive effect on others”.

### 3.4. Quality of Life

The patient’s subjective loss of quality of life in the last week, analysed through the visual analogue uniscale (VAS), indicated an average impact of 6.95 out of 10 (with a range from 3 to 10, and a standard deviation of 1.638). The (Spearman) correlations between the concerns and the loss of quality of life were positive in all cases, with the themes “Having unfinished business” (r = 0.36, *p* = 0.018) and “Fear of being forgotten” (r = 0.39, *p* = 0.057) being the highest in value.

## 4. Discussion

Few studies have investigated the subjective needs of patients with advanced cancer and their family members’ perception of the patients’ needs [8]. That is important for mainly two reasons: first, patients often cannot communicate their needs efficiently at the end of their life and need their relatives for this communication. Second, this information is needed to improve the palliative care service at the end of the life. Our study highlights the concerns of the terminally ill patients with insomnia, the perception of relatives regarding what they believe worries the patient, and the comparison of both. We found that 87.5% of the patients stated having nocturnal concerns which generated difficulties in falling or staying asleep. This result is considerably higher than the results of a prior study in the United Kingdom [8] but similar to the observed results in a previous Spanish study carried out in a similar setting [33], and is coherent with the importance of rumination identified in the study of Galfin and Watkins [4]. Our results predominate existential and personal concerns, which also fit the Galfin and Watkins ones [4].

Relatives only partly perceive the patient’s concerns. Patients and family members consider that the possible suffering and what will happen to the family afterwards to be the most worrying, but patients express more concerns and suffer them more intensely than what their relatives believe, except regarding two concerns: “what will happen to my family afterwards” and “having lived life to the fullest”. On the other hand, noticeably in three out of nine concerns analysed, there was no significant correlation between the intensity of the patient’s worry, and that which the relative perceives. These are fundamentally spiritual concerns: “dying alone”, “being able to say goodbye” and “having had a positive effect on others”. In all cases, relatives underestimate the presence and intensity of these concerns. This seems to indicate a significant lack of communication between patients and relatives concerning more abstract or transcendental themes. The lack of concordance of the perceptions of the relatives about the patients’ worries and concerns fits to the study of Oi-Ling et al.—caregivers and professionals failed to rate the most distressful symptoms in agreement with the patients [25]. Furthermore, in contrast to other studies which detected important needs for medical information [10,39], patients seem to have their basic information needs covered: only five patients indicated having had little medical information.

The qualitative study carried out from interviewing patients indicates that personal concerns are most frequent, especially the concern over the loss of control and autonomy. Overall, in this situation of proximity to death, patients report a loss of quality of life in the last week of almost 70%, similar to the results of the pioneering study of Morris et al. [40], where about 20% of the patients did not fall into the extremely low quality of life categories, even in the week prior to death. An important aspect regarding quality of life has also been detected: patients’ concern over having unfinished business, which portrayed a strong correlation value with the subjective loss of quality of life. This concern, once identified, should be worked at with the family for the patient to feel better, and palliative care professionals may also contribute to this, once these divergences have been noted in specific cases.

The study is not without limitations. First, the concerns proposed in the questionnaire were limited (nine items) so as not to tire out the patient and avoid emotional impact, which could be generated from an excessive number of questions. This limitation was partly counterbalanced through the semi-structured interview (open questions about possible concerns in four areas), given that patients showed significant differences in the quantity of details when talking about their worries. Second, the methodology used on a qualitative level implies limitations when generalising results, as it aims to reach a theoretical representativity, and not a statistical one. We believe that it would be appropriate to be able to expand the sample and incorporate patients in different settings, including a group of patients at home in their final stages of life.

This study completes previous ones [1,15,18,20], by contributing to a unified vision of the patient’s concerns from both the patient and the relative’s perspectives. Knowing the concerns of the oncological palliative patient with insomnia from both perspectives is crucial in providing the best possible attention in these situations where life expectancy is short, needs are constantly changing, and the patients are more worried than their relatives are aware of. Furthermore, our results support the recommendation to develop specific interventions, aimed at reducing the worries of an advanced patient with insomnia [33]. As can be seen from the present investigation, a very promising course of action would encourage the predisposition to actively listen and enhance communication with family members.

## 5. Conclusions

In conclusion, knowing convergencies and divergencies of the patient’s concerns by the relatives can provide valuable information for specific therapeutic interventions that can reduce the worries of the advanced patient with insomnia and ameliorate the quality of life and the communication in the end of life.

## Figures and Tables

**Table 1:** Medical and sociodemographic data (MSD) of the patient (*N* = 63).

MSD	Category	*N*	Percentage (%)
Gender	Men	43	68.3
	Women	20	31.7
Age	Median	74.7	
	Standard deviation	11.7	
Origin	Home	50	79.4
	Acute care	4	6.4
	Others	8	13.2
Tumour localisation	Lungs	14	22.2
	Urinary bladder	10	15.9
	Colon	9	14.3
	Myeloid leukaemia	5	7.9
	Stomach	5	7.9
	Hepatic	4	6.4
	Prostate	4	6.4
	Ovaries	4	6.4
	Breast	4	6.4
	Brain	1	1.6
	Pancreas	1	1.6
	Melanoma	1	1.6
	Not specified	1	1.6
Tumour extension	Dissemination	51	81
	Regional	8	12.6
	Local	3	4.8
	Unknown	1	1.6
Effect on the CNS	No	57	90.5
	Yes	6	9.5
Onset of insomnia	Before diagnosis	25	39.7
	Since diagnosis	25	39.7
	After entering hospital	13	20.6

**Table 2:** Answers obtained in the questionnaire of the patient’s concerns (according to the patient and the relative).

	According to the	Patient (*n* = 63)	According to the	Relative (*n* = 53)
Concerns	*n* of patients (percentage)	Average intensity of the concern	*N* of patients (percentage)	Average intensity of the concern
		(1–10)		(1–10)
Not knowing what happens after death	53 (84.1)	7.8	28 (52.8)	7.3
What happens to my family afterwards	58 (92)	8.4	50 (94.3)	8.4
Fear of being forgotten	53 (84.1)	8.2	19 (35.8)	4.5
Having unfinished business	63 (100)	7.8	36 (68)	7.3
The possible suffering during the process	59 (93.6)	9.3	41 (77.4)	9.2
The possibility of dying alone	57 (90.5)	7.8	39 (73.6)	6.7
Being able to say goodbye to loved ones	56 (88.8)	8	24 (45.3)	7.5
Having had a positive effect on others	53 (84.1)	7.2	43 (81.1)	6.1
Having lived life to the fullest	56 (88.8)	7.1	53 (100)	6.8

**Table 3:** Patient concerns (*n* = 63) expressed in the open interview, grouped into categories (*n* = number of participants that show concern, percentage in relation to the total amount of interviewed patients).

Category	*N*	Percentage (%)
Personal		
The loss of autonomy	59	93.7
Suffering	42	66.7
Experiencing pain	38	60.3
Lose control of the situation	35	55.5
Feeling physically weak	22	35
Having little medical information	5	7.9
Family		
How will my family cope the loss	26	41.3
Creating suffering in the family	20	31.2
Not being able to take care of the family	12	19.0
Being a burden to the family	6	9.5
Family conflicts	6	9.5
Not being able to speak to them about the illness	3	4.8
Spiritual		
The need to love and being loved	12	19
Forgiving and being forgiven	7	11.1
Being able to share	6	9.5
Being able to reconcile with the family	6	9.5
Being able to review one’s life properly	5	7.9
Feeling guilty	4	6.3
Find a meaning	4	6.3
Reencountering someone lost	2	3.2
Economic		
The current financial situation	12	19
Unfinished economic issues	8	12.7
Losing autonomy in decision-making	5	7.9
What I leave to my family	3	4.8
Being able to pay complementary therapies	2	3.2

## Data Availability

The data are available on request.
